# The Clinical Application Value of Multiple Combination Food Intolerance Testing

**Published:** 2019-06

**Authors:** Shudong LIN, Xiujing YANG, Ying XING, Xingye WANG, Yadong LI

**Affiliations:** Clinical Laboratory, The Third Affiliated Hospital of Qiqihar Medical University, Qiqihar, P.R. China

**Keywords:** Food intolerance, Immunoglobulin G, Value, Disease

## Abstract

**Background:**

We aimed to investigate the clinical value of detecting 14 food intolerances.

**Methods:**

A total of 312 patients with food intolerance enrolled in the Third Affiliated Hospital of Qiqihar Medical University (Qiqihar, China) from Feb 2016 to Feb 2017 were selected. ELISA was used to detect intolerance specific IgG antibodies for 14 foods (pork, chicken, beef, shrimp, fish, crab, egg white/yolk, tomato, mushroom, milk, corn, rice, soybean, wheat).

**Results:**

The highest average positive rate of the patients was 42.31% for the crab, followed by shrimp 21.15%, egg white/yolk 18.27% and milk 16.99%. The positive rate from high to low was crab, shrimp, egg white/yolk, milk, fish, corn, soybeans, tomatoes, rice, mushrooms, wheat, pork, beef, chicken. There were significant differences in the specific IgG antibody positive rates between shrimp, soybean and wheat in the skin symptoms group, gastrointestinal symptoms group, respiratory symptom group and nervous system symptom group (*P*<0.05). There was a significant difference in the positive rate of specific IgG antibodies between shrimp, crab and egg white/yolk in the adolescent group, the middle-aged group and the elderly group (*P*<0.05).

**Conclusion:**

The detection of food-specific IgG antibodies can help to determine which food intolerance caused the disease, and then adopt a fasting or diet method to avoid eating unsuitable foods and continually damaging the body, thus maintaining good health. The detection method provides a new idea for the diagnosis and prevention of diseases.

## Introduction

With the rapid development of our society and the improvement of living standards, the health of the diet has received more and more attention. People are not only paying attention to the nutritional balance, but also paying more attention to whether food is beneficial to people’s bodies. Food intolerance is an allergic disease mediated by immunoglobulin G (IgG). When the body ingests intolerant food, the specific IgG antibody produced by the immune system combines with the allergen in food to form immunity complex, causing varying reactions of the body, involving the digestive system, respiratory system and skin (1). Food intolerance is becoming more and more common with an incidence rate of 20% to 45%, which has become one of the hottest issues in research at home and abroad (2). At present, the diagnosis of food intolerance mainly depends on medical history, double-blind placebo-controlled trials and *in vitro* allergen-specific immunoglobulin G (sIgG) detection. Among them, serum sIgG detection has high flux and simple operation, which has extensive clinical application (3). Food-specific IgG was detected by food intolerance to find foods that are intolerant, so as to find out the real cause of the disease. Then a food restriction plan is developed to guide patients to avoid ingesting intolerant foods using fasting or less intolerant foods.

With the control on the source of the disease, preventing the development of the disease will be prevented till the disease is gone, and the quality of life will be improved significantly. This study used a new combination of four food intolerances launched by HOB to investigate the food intolerance of the corresponding population clinically, and to analyze the causes of the disease, in order to provide a basis for the diagnosis and treatment of the disease.

## Methods

### Clinical data

Overall, 312 patients who received food intolerance test in the Third Affiliated Hospital of Qiqihar Medical University (Qiqihar, China) from February 2016 to February 2017 were selected as subjects. 1) Gender was not limited, aged 0∼14 years old; 2) There were 14 kinds of foods in the supplementary food (pork, chicken, beef, shrimp, fish, crab, egg white/yolk, tomato, mushroom, milk, corn, rice, soybean, wheat); 3) informed consent. Exclusion criteria: 1) those who had recent history of plasma or blood transfusion; 2) those who received immunosuppressants, hormones or desensitization before enrollment. There were 186 males and 126 females; aged 8 to 82 years old, average (36.2 ± 22.5) years old. According to the symptoms, there were 78 cases in the skin symptom group, 102 cases in the gastrointestinal symptoms group, 82 cases in the respiratory symptoms group and 50 cases in the neurological symptom group. According to the age, there were 56 cases in the adolescent group, 206 cases in the middle-aged group and 50 cases in the elderly group.

This study was approved by the Ethics Committee of the Third Affiliated Hospital of Qiqihar Medical University.

### Reagents and test methods

HOB’s food-specific IgG antibody detection kit was produced by Shenzhen Boca Biotechnology Co., Ltd., and 14 food-specific IgG antibodies in human serum were detected by ELISA. The microplate reader was from AWARENESS, USA.

### Specimen acquisition

In the morning, 2 ml of venous blood was collected using a vacuum blood collection glass tube containing 7.5% EDTA-2Na 30 μL and April otinin 40 μL, allowed to stand at room temperature for 30 min, and centrifuged at (4000 rpm × 5 min) to obtain serum. Serum was stored in a refrigerator at −80 °C for subsequent use.

### Detection method

A standard curve with a linear range of 40, 80, 160, 320, 640 U/ml was illustrated; the serum was thawed and diluted100 times, 100 μl of the serum was added to each well, incubated for 1 h at room temperature; the plate was washed and dried, 100 μl of anti-human IgG antibody plus horseradish peroxidase binding solution was added, incubated for 0.5 hour at room temperature; the plate was washed and dried, added with 100 μl substrate mixture, incubated for 10 min at room temperature, added 50 μl stop solution, mixed well and the absorbance was measured at 450 nm.

### Result determination

The standard curve was drawn by taking the absorbance as the ordinate and the concentration as the abscissa. Concentration <50 U/ml was a negative standard; 50∼100 U/ml was a mildly sensitive standard; 100∼200 U/ml was a moderately sensitive standard; >200 U/ml was a highly sensitive standard.

### Statistical analysis

All data were analyzed using SPSS 20.0 (Chicago, IL, USA). The positive rate was expressed as a percentage and analyzed using the χ2 test, and the average antibody concentration was expressed as (χ¯±s) and tested by t-test. *P<*0.05 for the difference was statistically significant.

## Results

### Overall inspection

The average positive rate of the patient group was the highest, 42.31%, for the crab, followed by shrimp 21.15%, egg white/yolk 18.27%, and milk 16.99%. The positive rate from high to low was crab, shrimp, egg white/yolk, milk, fish, corn, soybeans, tomatoes, rice, mushrooms, wheat, pork, beef, and chicken (Table 1).

**Table 1:** Fourteen food-specific IgG antibody levels

***Antigen***	***Negative***		***Mildly sensitive***		***Moderately sensitive***		***Severely sensitive***		***Positive***		***Average of antibody concentration (U/ml)***
***Viarable***	***Number of cases***	***%***	***Number of cases***	***%***	***Number of cases***	***%***	***Number of cases***	***%***	***Number of cases***	***%***	
Pork	308	98.72	3	0.96	1	0.32	0	0.00	4	1.28	102.3±5.6
Chicken	311	99.68	1	0.32	0	0.00	0	0.00	1	0.32	72.6±6.3
Beef	310	99.36	2	0.64	0	0.00	0	0.00	2	0.64	65.3±15.2
Shrimp	246	78.85	36	11.54	22	7.05	8	2.56	66	21.15	66.8±12.5
Fish	283	90.71	22	7.05	5	1.60	2	0.64	29	9.29	89.6±11.5
Crab	180	57.69	112	35.90	16	5.13	4	1.28	132	42.31	85.3±16.3
Egg white/yolk	255	81.73	22	7.05	15	4.81	20	6.41	57	18.27	165.3±35.7
Tomato	294	94.23	10	3.21	4	1.28	4	1.28	18	5.77	102.4±32.1
Mushroom	296	94.87	12	3.85	2	0.64	2	0.64	16	5.13	90.2±11.5
Milk	259	83.01	30	9.62	13	4.17	10	3.21	53	16.99	156.9±37.9
Corn	286	91.67	20	6.41	5	1.60	1	0.32	26	8.33	80.2±12.5
Rice	294	94.23	15	4.81	2	0.64	1	0.32	18	5.77	91.5±12.6
Soybeans	293	93.91	16	5.13	3	0.96	0	0.00	19	6.09	86.3±9.5
Wheat	299	95.83	13	4.17	0	0.00	0	0.00	13	4.17	75.1±8.6

### Comparison of food intolerance in patients with different symptoms

There were significant differences in the specific IgG antibody positive rates among shrimp, soybean and wheat in the skin symptoms group, gastrointestinal symptoms group, respiratory symptom group and nervous system symptom group (*P*<0.05) (Table 2).

**Table 2:** Comparison of 14 food-specific IgG antibody positive rates in patients with different symptom

***Antigen***	***skin symptoms group(n=78)***		***gastrointestinal symptoms group(n=102)***		***respiratory symptom group(n=82)***		***nervous system symptom group(n=50)***	

	***Number of cases***	***%***	***Number of cases***	***%***	***Number of cases***	***%***	***Number of cases***	***%***
Pork	1	1.28	1	0.98	1	1.22	1	2.00
Chicken	0	0.00	1	0.98	0	0.00	0	0.00
Beef	1	1.28	0	0.00	1	1.22	0	0.00
Shrimp^*^	10	12.82	26	25.49	17	20.73	13	26.00
Fish	7	8.97	11	10.78	9	10.98	2	4.00
Crab	33	42.31	41	40.20	34	41.46	24	48.00
Egg white/yolk	14	17.95	18	17.65	15	18.29	10	20.00
Tomato	4	5.13	5	4.90	5	6.10	4	8.00
Mushroom	4	5.13	6	5.88	3	3.66	3	6.00
Milk	15	19.23	17	16.67	16	19.51	5	10.00
Corn	7	8.97	10	9.80	8	9.76	1	2.00
Rice	4	5.13	5	4.90	4	4.88	5	10.00
Soybeans^*^	2	2.56	6	5.88	7	8.54	4	8.00
Wheat^*^	1	1.28	1	0.98	8	9.76	3	6.00

* *P*<0.05

### Comparison of food intolerance among patients of different ages

There was a significant difference in the positive rate of specific IgG antibodies among shrimp, crab and egg white/yolk in the adolescent group, the middle-aged group and the elderly group (*P*<0.05) (Table 3).

**Table 3:** Comparison of 14 food-specific IgG antibody positive rates among patients of different ages

***Antigen***	***Adolescent group(n=56)***		***Middle-aged group(n=206)***		***Elderly group(n=50)***	
	***Number of cases***	***%***	***Number of cases***	***%***	***Number of cases***	***%***
Pork	1	1.79	2	0.97	1	2.00
Chicken	0	0.00	1	0.49	0	0.00
Beef	1	1.79	1	0.49	0	0.00
Shrimp^1)^	8	14.29	42	20.39	16	32.00
Fish	8	14.29	10	4.85	11	22.00
Crab^2)^	40	71.43	89	43.20	3	6.00
Egg white/yolk^3)^	22	39.29	29	14.08	6	12.00
Tomato	3	5.36	9	4.37	6	12.00
Mushroom	2	3.57	12	5.83	2	4.00
Milk	9	16.07	35	16.99	9	18.00
Corn	6	10.71	16	7.77	4	8.00
Rice	3	5.36	12	5.83	3	6.00
Soybeans	3	5.36	13	6.31	3	6.00
Wheat	2	3.57	9	4.37	2	4.00

1) *χ*^2^= 6.512, *P*=0.039;

2) χ^2^= 46.528, *P*<0.01;

3) χ^2^= 20.305, *P*<0.01

## Discussion

The cause of food intolerance is that when food enters the digestive tract, it can only be converted into energy for human body when digested into glycerol, amino acids and monosaccharides, but some foods cannot be completely digested due to lacked corresponding enzymes. Those foods exist as a polypeptide or other molecule, recognized as a foreign substance by the body, resulting in an immune response, producing food-specific IgG antibodies and binding to food molecules to form an immune complex, which can cause inflammation of tissues and organs, thereby manifesting as systematic symptoms and diseases (4–6). Therefore, the detection of food-specific IgG antibodies is of great significance for judging whether the human body has developed lesions due to food intolerance, it also provides a new idea for disease diagnosis.

Studies have shown that food intolerance is associated with genetic, long-term accumulation and growth environments, and the use of food additives, environmental pollution and food cooking methods can affect food intolerance (7, 8). Therefore, the results of food intolerance testing should be comprehensively analyzed. This study performed detection on 14 food intolerances in 312 patients with food intolerance in Qiqihar, and varying degrees of intolerance were found in various foods. Among them, the highest intolerance positive rate was found in crab, followed by shrimp, egg white/yolk and milk. The positive rate from high to low was crab, shrimp, egg white/yolk, milk, fish, corn, soybean, tomato, rice, mushroom, wheat, pork, beef, chicken. It can be seen that the population of the region was more intolerant to seafood and eggs, and the degree of intolerance to rice, corn, beef, chicken and food was low, the reasons were: 1) the region was inland, it was an important commodity grain base and animal husbandry base in China. It was far from the ocean and the population was poorly tolerant to seafood but highly tolerant to rice, mushrooms, wheat, pork, beef and chicken. 2)

Seafood and high-protein food were highly immunogenic, and most of the proteins were acidic isoelectric glycoproteins, which can tolerate food processing and resist intestinal digestion. For patients with positive food intolerance, conducting appropriate health education and psychological counseling, assessing their health status and nutritional needs, helping them adjust their diet and change long-term eating habits slowly were of great importance to change their disease status (9–11). The results of this study showed that there were significant differences in the positive rates of specific IgG antibodies among shrimp, soybean and wheat in the skin symptoms, gastrointestinal symptoms, respiratory symptoms and neurological symptoms groups (*P*<0.05), indicating that symptoms were related to food intolerance, and patients with different symptoms had different degrees of tolerance to shrimp, soybeans and wheat. The recurrent aphthous ulcer food intolerance specific IgG antibody positive rate test results showed that the first two among the positive rates were shrimp and crab, indicating that seafood may be a common antigen of digestive tract diseases (12–15). By dividing patients into different age groups, the results showed that adolescent patients had higher positive rates for shrimp, crab and egg white/yolk-specific IgG antibodies, which may be due to imperfect digestive system function in children and teenagers, resulting in more vulnerable of forming IgG antibody against the foreign protein in the intestine.

## Conclusion

In summary, the detection of food-specific IgG antibodies can help to initially determine the disease caused by which food intolerance, and adopt a fasting or diet method to avoid eating unsuitable foods continuously damaging the body, thus maintaining good health, providing a new idea for the diagnosis and prevention of diseases.

## Ethical considerations

Ethical issues (Including plagiarism, informed consent, misconduct, data fabrication and/or falsification, double publication and/or submission, redundancy, etc.) have been completely observed by the authors.
